# On the Proximate Causes of Diseases, and Particularly of Fevers, in Continuation of His Letters on Quarantines, &c.

**Published:** 1809-02

**Authors:** Thomas Alder


### *On the proximate Causes of Diseases, and particularly of Fevers, in continuation of his Letters on Quarantines, &c.* By Dr. ALDER.

I Fear it will be utterly impossible to give any who have a contempt for *reasoning*, or at *my attempts at reasoning*, any sort of pleasure in reading these letters: for though *the facts*, I trust, will be sufficient and authentic, so as to warrant my conclusions, yet deduction will be crammed upon deduction, and inference upon inference, so quick, that in order to preserve such gentlemen from the painful feelings of disgust, I think I shall do well to advertise them in the beginning, not to read one line farther.

I hope, however, it must appear to many, that *reason* is the noblest faculty which the Almighty has bestowed upon living creatures, and that he who makes the best use of it, must *(cæteris paribus)* be most acceptable to Him, and generally most serviceable to His creatures.

I allow that I do not think it is in my province to experiment, or to confine my attention solely or chiefly to the facts which fall under my own observation; and I conceive that it is my duty, in these investigations, to view all the facts connected with them *impartially*, (so far as it is in my power to do this) and then to make the best inferences which I can *from them all collectively*, and to take especial care not to admit false facts, or to be misled by or indulge in *mere conjectures*.

It appears to me incumbent on me too, to advance nothing but what *may be* understood by very plain capacities, (which for common purposes seem the best) possessing a liberal medical education; and to leave out not only what is unnecessary or collateral, but also that which is very intricate.—But on the other hand, I must acknowledge, that in order to make myself very clear, I cannot but fear I shall too much expose myself to the charges of dryness, tautology, and pedantry.

But

But to begin: I shall first notice a few universally admitted facts. 1st. All experiments prove the necessity for oxygen; that warm-blooded animals without it, die in a few minutes; *and that its action is upon the lungs.* These are very simple facts; but if we add to them, 2dly. oxygen stimulates the pulmonic vessels, and by that means makes them propel their contents, we shall have almost all the facts necessary to lead us to the proximate cause of most diseases.

For, consider, 1st. that *the oxygen, by stimulating the pulmonic vessels, makes them transmit the blood from the right side of the heart to the left, and from the veins into the arteries: and, 2dly, that when oxygen does not stimulate these vessels sufficiently, they will not without assistance from some other cause, sufficiently propel their contents, and transmit the blood from the veins into the arteries.*—I trust these simple facts and inferences are so obvious to all thinking men of our profession, that I shall for the present omit the proofs of them, in order to particularize a few more equally obvious deductions. When the blood is not duly propelled through the pulmonic vessels, it must *accumulate in the lungs;* 2dly, it must be *deficient in the arteries;* 3dly, it must be *superabundant in the veins.* But from these three circumstances, all the phænomena of the more dangerous and prevalent diseases can be traced *with ease.* For, 1st. *when blood is accumulated in the vessels of the lungs,* the breathing will be difficult or laborious, as it is in most acute diseases; and there will be a most anxious and distressing feeling (in general) about the chest; at first, often with some chilliness, (the vessels containing the pulmonic congestion, at first being checked in their action) but in a short time there will be a glow in the chest, and sometimes quickly after, considerable heat and pain; for the congested vessels will not *in general* be long before they are stimulated to re-action by the quantity of blood distending them, and sometimes their action will become so great, as to be *inflammation.*

We now come to consider, 2dly. *The blood will be deficient in the arteries,* when it is *first* retarded and accumulated in the lungs. Hence the pulse and muscular power, &c. will be surprisingly reduced in a remarkably short time, (sometimes in half an hour or a few minutes) and may be entirely taken away; which in acute diseases at the attack they often are.

3dly. We notice *the veins being full of blood at the same time,* but especially the large veins. It is evident the pulmonary monary arteries as they are called, and the venæ cavæ must very soon be overcharged and overdistended with blood, when this cannot pass freely from them through the lungs into the arteries; and after them the veins in the head, liver, spleen, stomach, and intestines, &c. in quick succession, will be overcharged, because the blood will still be pressed into them from the smaller veins, (more or less) and into the smaller veins from the arteries. And when the veins in all these organs are overcharged, (the arteries in them at the same time being depleted) the functions of these organs will be greatly disturbed; *just as we find they are in the attack of acute diseases.*—But though many, or all of these symptoms, in a greater or less degree, according to the intensity of their cause and other circumstances, must generally shew themselves when the blood is prevented from passing freely through the lungs, yet on some occasions some will preponderate, and on other occasions others. And when either the accumulation SUDDENLY causes *suffocation,* or *irritation,* or *inflammation,* very few have *time* to shew themselves; hence they are not all well-marked in several instances of fever, pneumonia, or suffocation; and when the veins in the head are soon overcharged, or the brain is tender or irritable, all these symptoms too are not well seen, for apoplexy or convulsions attack.

But if we recur to the congestion of blood as first formed in the lungs, we may readily anticipate it may destroy by suffocation, by syncope, by apoplexy, or by epilepsy; we may see too it may cause polypi, or ruptures, &c. in the large veins; it may cause hæmoptysis, or a great secretion of mucus, or serum; as well as bring on irritation, or inflammation in the pulmonic vessels, and then by them great increased action of the arteries. And we may discern that hæmoptysis (and some of the other consequences) is most likely to happen just when the congested vessels are coming to the height of re-action; and that epilepsy and apoplexy are most liable to occur when the blood has been driven violently into the arteries; as the arterial system in the head will just at that time be filled, while the venous is not yet depleted.—We may foresee also, that the liver, spleen, stomach, and intestines, &c. will in like manner be most liable to violence of inflammation precisely at the same time, and for the same reasons, as also any of the inferior parts, (as the eyes, the ears, the nose, the hæmorroidal vessels, &c.) all which, from local causes, *occasionally* become the most conspicuous seats of venous congestions.

But

But these simple deductions are most fully confirmed to be just by the history of diseases, by the treatment of them, and by anatomy, as we shall now see, or as the greater part of practitioners (I may rather say) must have seen as they read the preceding.

*Proofs of the preceding Theory of the proximate Causes of Diseases; and first from the History of Diseases.*

In the short compass of a few letters, it is not to be expected that I can notice the whole of the symptoms which confirm what has preceded, or dwell long upon any of them. I shall begin with fevers, as they in their bad kinds are a compend of almost all the other diseases. In the beginning of most fevers, there is every symptom of the pulmonic vessels being overloaded with blood, without being irritated into re-action by the distension of them; great oppression and anxiety about the chest; most difficult but *cold* respiration; and a very small feeble pulse. Bye and bye (as the second stage comes on) we find every mark of this congestion in the lungs becoming active: faint heat is perceived about the chest; the respiration is more warm; the arteries become better supplied: we sometimes find too that after a little re-action of these over-distended pulmonic vessels, they generally go forwards re-acting, till they propel the blood into the arteries as fast as it comes into them, (from their irritated state) but do not for some time recover their natural tone; hence there is a laborious respiration for some time after they have begun to re-act. Sometimes they subside to their natural tone after violently propelling their contents for a few hours; in which case the fever ceases for a time, or remits; but returns from the causes which first occasioned it: sometimes the irritation is kept up for two or three weeks, subsiding gradually; and sometimes they actually become inflamed; in most of the bad epidemic fevers, the lungs have become inflamed. But sometimes they make several *efforts* to drive forward their superabundant quantity of blood before they can succeed. The alternate chills and heats in the accessions of many of the common fevers of this country prove this; and so do the slight and treacherous remissions of the worse fevers of more unhealthy places; or of epidemic seasons.

But in short, all the symptoms of the common kinds of fever, or of the worst, can be so clearly traced, either from the passive or active congestion in the lungs, the deficiency of blood in the arteries, or the superabundance of it in the larger veins, and particularly those of the head, liver, and intestinal canal, or from a combination of these morbid states of the system, *and consequently so fully prove the existence of these respective morbid states of the system in fever,* that I hope I need not say much more upon the subject. I shall notice, therefore, only a few of those symptoms which occur in the worst kinds of fever, and which have seemed most inexplicable. As the great variations in the state of the system, which constitute *the stages* of fever, most fully prove my Theory of the inmost nature (proximate cause) of this complaint to be true; so do likewise the almost innumerable varieties in the *form of fever*: from my Theory, we must indeed see that *the varieties* of fever may be almost endless, and medical reading will shew they are so. Nosological writers have now distinguished, I think, near a hundred forms of fever, and they have not particularized, and indeed cannot particularize, nearly the whole of them yet.

There are, however, certain bad symptoms, which are common to almost all fevers, (varying only in degree) and some which are common to almost all bad fevers, whether epidemic or endemic, but which also vary in degree.—The affection of the respiration and chest, the sudden failure of the pulse and muscular power, and the complaints of the head, liver, and intestinal canal, are common to *most fevers,* and vary *chiefly* in degree. The immensely great and sudden failure of the pulse and muscular power, and prodigious anxiety about the chest, are usual characteristics of the attack of bad fevers; and a sudden turgescence in the veins of the head, (sometimes causing apoplexy on the first attack) great congestions in the lungs, liver, and intestinal canal, quickly attended with great heat and pain in these organs, are symptoms which are also common to *most bad fevers.* And as none of these symptoms admit of any other origin being assigned them than what the preceding Theory points out; and as the whole of them can be most clearly seen to spring from the proximate cause which I have assigned to fever, I think I may bring them as most unequivocal proofs of my Theory.

*Most* of these bad fevers (as well as some diseases which have not yet been made to come under the denomination of fever) have been called "*the plague,*" and so far as this term signifies a very distressing complaint, they have rightly been made to do so. But as there is almost nothing exactly in common in those diseases which have been called "*the plague,*" (as all of them have not, indeed, according to our present distinction of diseases, been entitled to be called called fevers) so also many other complaints, which have not been called *"the plague,"* have been as well entitled to be called so as those have. *"The plague"* is a vague, indefinite term, and ought to be exploded *from medicine.* It has usually been given to bad epidemic disease, but not always. A mild or a tolerable epidemic disease, and a severe endemic disease, even though a fever; and, (what is more) exactly like many of those fevers which have been called *"the plague,"* has seldom been called *"the plague."* A severe epidemic hæmorrhois, erysipelas, pneumonia, apoplexy, remitting fever, intermitting fever, and continued fever, &c. have respectively often been called *"the plague."* While, on the other hand, a mild, or not very rife, scarlatina, or influenza, &c. have not been called *"the plague,"* merely because they were moderate; and the fevers of Bengal, Batavia, and our unhealthy settlements in Africa, &c. have not been called *"the plague,"* (however bad they may have been) merely because they were owing to a well-known local cause, that is *miasma,* or impure air. But I trust I have shewn (and Mr. Noah Webster has done it much more fully than I have) that the sources of bad air are such as to be capable of causing (though no doubt they will often be assisted by other agents) all these fevers and diseases, whether epidemic, endemic, or sporadic. I therefore, in speaking of those symptoms which are common in most bad fevers, include all fevers, whether epidemic or endemic, and whether called *"the plague,"* or not. The common autumnal fevers of America, when very bad, and the common fevers of the West Indies, when very bad, (all which clearly spring from a vitiated atmosphere) exhibit the same symptoms in general which the bad epidemic fevers of those places do: so also do the endemics of Egypt when very bad, for the most part, shew the same symptoms as the epidemics of that country; but as the epidemics are often caused by a junction of the epidemic bad air with the endemic, so they often are more rife and more mortal; and as the epidemic bad air often has not nearly so much moisture in it as the endemic, or is in some other way different from it, the symptoms of the epidemic fevers sometimes somewhat differ from those of the endemic. But, *in general,* they all agree in the symptoms which I have stated.—And this being the case, I think I am warranted to say, *they all arise from a* SIMILAR *cause, and from one which induces congestions in the lungs.*

When *"the plague"* first appears, it sometimes kills, and particularly in Egypt, by apoplexy joined with phre-

 nitis, nitis, in half an hour. This is owing to the sudden invasion of very concentrated bad air upon constitutions previously disposed to apoplexy. Again, in the course of the worst fevers, there usually is a peculiar sparkling lustre in the eyes, with a most dejected and distressed countenance; and this has by some been said to be characteristic of "*the plague,*" but it occurs in many instances of the bad endemic fevers. It is owing to the venous system in the head being overcharged, so far as regards the great lustre in the eyes, and stimulating the brain almost to phrenitis. But the great dejection and distress are owing to the accumulation in the lungs. Two other symptoms too are mentioned, and especially by ancient writers, as greatly characterizing "*the plague:*" these are a prodigious burning heat with pain in the abdomen and chest, and *jactatio,* or a furious and incessant tossing about: the latter is a consequence of the former; all the viscera in the abdomen and chest have then had their veins especially, or their whole substance, wonderfully congested with blood, inflamed, and soon mortified, even when the arteries for the most part had been ill-supplied, and had not indicated any inflammation by increase of heat and action, but the patient on the contrary had been sinking under great debility and depression. These congestions, in such cases, have always been found very enormous by dissection; and the veins have always been found particularly gorged with black blood, and inflamed or mortified like the viscera. This latter circumstance, together with the feebleness of arterial action, and the depression and weakness, prove incontrovertibly that an obstructed passage of the blood from the veins into the arteries at the lungs, was the cause of those two violent symptoms which usually have been the most frightful of all that have occurred in bad endemics and epidemics, and which, (though erroneously) have been alleged to distinguish "*the plague,*" from diseases which have not obtained that denomination.

In my next I intend to pass on to the *anatomical proofs* of the Theory which I have advanced, and then to *others.*

I am, &c.

THOMAS ALDER, M. D.

*November* 29, 1808.

( To be continued. )

